# Risk of common infections among individuals with psoriasis in Sweden: A nationwide cohort study comparing secukinumab to ustekinumab

**DOI:** 10.1002/pds.5132

**Published:** 2020-09-25

**Authors:** Chaitra Srinivas, Ingvild Odsbu, Marie Linder

**Affiliations:** ^1^ Centre for Pharmacoepidemiology, Department of Medicine Karolinska Institutet Solna Sweden

**Keywords:** biologic drugs, cohort study, infections, pharmacoepidemiology, population register, psoriasis

## Abstract

**Purpose:**

To determine risk of respiratory tract infections, urinary tract infections and candidiasis in secukinumab users compared to ustekinumab users among individuals with psoriasis in Sweden.

**Methods:**

This was a Swedish population‐based register‐linked new‐user cohort study on individuals with psoriasis and psoriasis arthritis treated with secukinumab (2015‐2017) and ustekinumab (2009‐2017). Ever‐never exposure definition was used, that is, each individual's follow‐up time was attributed to the drug they were first exposed to. Risk of severe respiratory and urinary tract infections and candidiasis (diagnosis codes from out‐patient specialist visits and in‐patient hospitalisations) and respiratory and urinary tract infections treated in primary care (proxied by dispensation of antibiotics) was determined by adjusted hazard ratios (HRs) and 95% confidence intervals (CIs) using Cox regression. We also give crude incidence rates and rate ratios.

**Results:**

In total, 1955 new users of secukinumab (n = 848) and ustekinumab (n = 1107) were identified. There was a slightly increased risk of respiratory and urinary tract infections treated in primary care among secukinumab users compared to ustekinumab users (HR: 1.22, 95% CI: 1.03‐1.43). Non‐significant differences in estimated risk of severe respiratory and urinary tract infections (HR: 0.96, 95% CI: 0.57‐1.61) and candidiasis (HR: 1.80, 95% CI: 0.84‐3.84) treated in the hospital setting were observed.

**Conclusion:**

We observed a slightly increased risk of respiratory and urinary tract infections treated in primary care among secukinumab users compared to ustekinumab users. Larger studies with longer follow‐up are needed to draw conclusions on relative safety.

**Key points**

- Biologics secukinumab and ustekinumab are effective for psoriasis management. However, clinical trials of these biologicals highlight safety concerns such as respiratory tract infections, urinary tract infections and candidiasis.
- Health and population data from Swedish national registers provide a unique opportunity to investigate the safety of biologics in a real‐world setting.
- A slight increased risk of respiratory tract infections and urinary tract infections treated in primary care was observed among secukinumab users compared to ustekinumab users.
- Although both immune dysregulation and interleukin‐antagonist therapy can raise the risk of Candida infections, our findings showed no remarkable risk of candidiasis among secukinumab users compared to ustekinumab users.

## 1 INTRODUCTION

Psoriasis is a complex, immune‐mediated, chronic inflammatory disease affecting the skin, nails and joints.^1^, ^2^ Globally, psoriasis prevalence ranges between 0.09% and 11.4% among different populations.^3^, ^4^ In the Nordic region, psoriasis prevalence rate among adults is between 1% and 5%,^5^, ^6^ which equals from 100 000 to 500 000 individuals with psoriasis in Sweden. There is limited understanding of psoriasis aetiology.^7^ Genetic, environmental and immunologic factors are known to play a role in the complex pathogenesis of psoriasis.^3^ Also, external factors such as stress, infections and certain medications such as beta blockers and antimalarial drugs are known to aggravate the disease.^7^

Exploring the function of T helper 17 cells in the inflammatory cascade has resulted in the identification of specific antibody treatments for multiple immune‐mediated diseases including psoriasis.^8^ Biologics offer target‐specific immunosuppressive activity, improved safety profiles and better tolerability compared to traditional anti‐inflammatory medications.^7^, ^9^, ^10^ However, as biologics are inherently meant to act upon the immune system, they have the potential to cause other immune mediated effects besides their targeted action, including an increased susceptibility for infections.^11^, ^12^

Currently, interleukin (IL)‐inhibitors ustekinumab and secukinumab are being favoured in psoriasis management, as they have demonstrated improved skin clearance compared to their predecessor tumour necrosis factor‐α (TNF‐α) inhibitors etanercept and infliximab.^9^, ^13^, ^14^, ^15^, ^16^, ^17^ However, the implications of the long‐term activity of IL‐inhibitors outside of clinical trials is yet to be explored.^18^, ^19^, ^20^, ^21^ Secukinumab and ustekinumab act by targeting proinflammatory cytokines IL‐17A and IL‐12/23 respectively.^18^, ^22^ IL‐17A is involved in host immune defence, with roles in granulopoiesis, neutrophil trafficking and mucocutaneous defence against fungi and bacteria; thusly, raising concerns with anti‐IL‐17A therapy and its potential for infections.^22^ Risk of infections is also a concern among patients on anti‐IL12/23 therapy, since genetic deficiencies of IL‐12/23 can raise the risk of certain bacterial infections.^18^ The European Medicines Agency (EMA) risk management plans (RMP) of both secukinumab and ustekinumab highlights the risk of infections, upper respiratory tract infections (RTI) in particular for secukinumab use.^23^, ^24^, ^25^ Also, IL‐related immune protection has been evidenced, especially against fungal infections caused by *Candida* species. Therefore, anti‐IL therapy, puts patients at an increased risk of *Candida* infections due to their mode of action.^26^, ^27^, ^28^

Previous studies have shown conflicting results regarding clinical superiority of secukinumab over ustekinumab with respect to achieving desired clinical endpoints, demonstrating higher efficacy and safety.^9^, ^13^, ^29^ In a long‐term study exploring the safety and efficacy of biologics in psoriasis, secukinumab had more adverse events compared to other agents including ustekinumab.^13^ In a multicentre, randomised, double‐blinded clinical trial, secukinumab demonstrated superior clinical efficacy with improved quality of life when compared to ustekinumab and showed a comparable safety profile with ustekinumab.^29^ Since secukinumab is a relatively new drug, long‐term surveillance data is lacking.^18^, ^19^, ^20^, ^21^ Also, studies comparing the safety profiles of both ustekinumab and secukinumab are limited.^13^, ^29^

Clinical trials are carried out on select populations with short follow‐up not mirroring the clinical reality where the drugs are used once marketed.^30^ Continuous post‐marketing safety surveillance of drugs is crucial to capture clinical endpoints which are missed in conventional pre‐market clinical trials.^30^ The availability of register‐linked information in Sweden provides a unique possibility of obtaining effective insights into safety comparisons between drugs. This study aimed at determining the risk of RTI and urinary tract infections (UTI) for secukinumab use compared to ustekinumab use among individuals with psoriasis, using population data on clinical diagnoses and dispensed antibiotic prescriptions used as proxies for infections treated in primary care from Swedish national registers. As a secondary aim, the risk of candidiasis was investigated using population data on clinical diagnoses.

## 2 METHODS

### 2.1 Data sources

This was a nationwide population‐based cohort study conducted using data linked from multiple Swedish national registers – National Patient Register (NPR), Swedish Prescribed Drug Register (PDR), Swedish Cancer Register (SCR), Cause of Death Register (CDR), and population registers in Sweden.^31^, ^32^, ^33^, ^34^, ^35^, ^36^ The source population included all individuals registered in the period 1964‐2013 (closed cohort, see Appendix, Figure S1) with diagnosis codes for psoriasis and psoriasis arthritis coded using the International Classification of Diseases (ICD) which are – ICD‐10: L40, M070, M073 (from 1997); ICD‐9:696, 713D; ICD‐8:696; ICD‐7:706, recorded in the NPR. Records from both in‐patient care (from 1964) and out‐patient specialist care (from 2001) from NPR were included.^31^ Data on drug use was captured using the PDR. The PDR provides information on all dispensed prescriptions from pharmacies in the Swedish population.^32^ The drugs recorded in the PDR are coded using the Anatomical Therapeutic Chemical Classification System (ATC) index.^37^ Information on migration was obtained from population registers of Statistics Sweden.^35^, ^38^ The SCR was used to obtain morphologically verified cancer diagnoses which could be potential confounders in the study.^33^ The CDR was used to capture mortality data.^34^ Data up to December 31, 2017 were included for all the data sources except for data from Statistics Sweden where data up to December 31, 2016 were included.

### 2.2 Outcomes

The outcome measured was common infections (see Appendix, Table S2). This comprised of upper and lower RTI, UTI and candidiasis. The RMPs for secukinumab and ustekinumab highlight upper RTI as a common safety event.^24^, ^25^ UTI were added since they are a common group of infections especially in primary care.^39^ Additionally, we studied candidiasis, since patients with psoriasis are at an increased risk of fungal infections and anti‐IL therapies can further this risk.^26^, ^27^ RTI, UTI and candidiasis were identified as per: (a) ICD‐10 (tenth version of ICD) classifications captured from in‐patient and out‐patient specialist care data from the diagnosis records obtained from NPR. RTI and UTI were also identified as per: (b) Dispensed prescriptions for antibiotic use for RTI and UTI recorded in the PDR. Dispensed prescriptions were used as proxies to capture infections treated in primary care setting. The infections subtypes RTI and UTI based on diagnosis codes were grouped and evaluated as the outcome “severe RTI and UTI.” The infections subtypes RTI and UTI based on dispensed antibiotics were grouped and evaluated as the outcome “antibiotics for RTI and UTI.” Also, all the infection subtypes were evaluated separately.

### 2.3 Exposures

Secukinumab use (ATC code: L04AC10) and ustekinumab use (ATC code: L04AC05) were the exposures considered in this study. A new user was considered as the first‐time user of either study drug, that is, ustekinumab (2009‐2017) or secukinumab (2015‐2017). New user definition was based on the first prescription dispensed during the study period with no dispensed prescription of the drug within the last 12 months. All individuals who were new users of ustekinumab were considered as the reference group. Individuals who were new users of secukinumab were considered as the comparator group. Individuals were counted only as pure users of secukinumab or pure users of ustekinumab, that is, switching was ignored. Ever‐never exposure definition was used to handle all the drug exposures, that is, each individual's follow‐up time was attributed to the drug they were first exposed to during the study period. The follow‐up time period for each drug group began from the index date of treatment with either drug and ended on the date of the following: outcome (RTI, UTI, candidiasis), death, emigration or end of study (December 31, 2017), whichever occurred first. Each outcome was analysed separately.

### 2.4 Covariates

Information on sociodemographic covariates including age, sex, education, income, occupation, civil status and region, was obtained from NPR and population registers of Statistics Sweden.^31^, ^35^, ^38^ Clinical covariates related to both psoriasis and the exposure drugs were considered as potential confounders. All the covariates were included in the study based on existing knowledge from relevant scientific literature. Information on these clinical covariates was obtained from NPR and SCR. Sociodemographic covariates were assessed 1 year before index date and clinical covariates were assessed starting from 1997. Missingness is a minor problem in the Swedish national registers. If information on a variable, for example, diagnosis, was available, patients were coded as not having the diagnosis if that specific diagnosis code was not found. For other types of variables where “missing” was one of the possible values, for example, for geographical area, the category “missing” was used; geographical area can be “predominantly urban,” “intermediate,” “predominantly rural” or “missing.”

### 2.5 Statistical analysis

Baseline characteristics of the study population were expressed as numbers and proportions. Additionally, the number of events (RTI, UTI, candidiasis), incidence rates (IR), person‐time in days and incidence rate ratios (IRR) for all the study outcomes were tabulated. Cox proportional hazards models^40^ with time‐scale in person‐days were used to estimate the crude and adjusted HR, with index drug date as the start time for the follow‐up period. Two models were fitted to analyse the differences among the two exposure groups. Each model was fitted separately for each outcome. The crude model included only the exposure and the outcomes to determine the unadjusted association. The adjusted model was adjusted for sociodemographic variables – age, sex, education, income, partner (civil status), occupational status and region (county of residence) and clinical variables relevant to both exposures and outcomes – (psoriatic arthritis, cancer, immunocompromised status, diabetes, chronic obstructive pulmonary disease [COPD], renal diseases, tobacco use, radiation, bacterial infections, asthma) before index. The proportional‐hazards assumption was tested for the adjusted model (see Appendix, Table S4). To address the imbalance in market availability, a sensitivity analysis restricted to the first 3 years on the market for secukinumab (2015‐2017) and ustekinumab (2009‐2011) was added (see Appendix, Table S1). STATA version 14.0 (StataCorp, TX, USA) was used for all analyses.

## 3 RESULTS

The study cohort consisted of 1955 individuals diagnosed with psoriasis or psoriatic arthritis from NPR. While 43.4% (n = 848) of the population were index users of secukinumab between 2015 and 2017, 56.6% (n = 1107) of the population were index users of ustekinumab between 2009 and 2017 (see Appendix, Figure S2). The baseline descriptive sociodemographic and clinical characteristics of the study drug groups are presented in Table 1. Males were the majority in the ustekinumab group (54.6%), whereas females were the majority in the secukinumab group (51.8%). A large proportion of individuals belonged to the age group 30 to 69 years within both drug groups. While over 65% of secukinumab users had one or more comorbidities at baseline, over 58% of ustekinumab users had one or more comorbidities at baseline (see Appendix, Table S3 for detailed description of comorbidities).

**TABLE 1:** Baseline sociodemographic and clinical characteristics of individuals with psoriasis treated with secukinumab or ustekinumab (n, %) (N = 1955)

Characteristic	Secukinumab	Ustekinumab
Inclusion period (year)	2015–2017	2009–2017
Patients ^a^ (N = 1955)	848 (43.4%)	1107 (56.6%)
Sex		
Male	409 (48.2%)	604 (54.6%)
Female	439 (51.8%)	503 (45.4%)
Age		
10‐29 years	65 (7.7%)	115 (10.4%)
30‐49 years	332 (39.2%)	436 (39.4%)
50‐69 years	395 (46.6%)	486 (43.9%)
≥70 years	56 (6.6%)	70 (6.3%)
Education		
<9 years	133 (15.7%)	247 (22.3%)
9–12 years	445 (52.5%)	554 (50.0%)
>12 years	264 (31.1%)	290 (26.2%)
Missing values	6 (0.7%)	16 (1.4%)
Income ^b^ (in quartiles)		
Low	175 (20.6%)	313 (28.3%)
Mid low	214 (25.2%)	274 (24.7%)
Mid high	217 (25.6%)	271 (24.5%)
High	240 (28.3%)	247 (22.3%)
Missing values	2 (0.2%)	2 (0.2%)
Partner ^c^		
Yes	392 (46.2%)	451 (40.7%)
No	456 (53.8%)	655 (59.2%)
Missing values	0 (0.0%)	1 (0.1%)
Occupational status ^c^		
Employed	586 (69.1%)	706 (63.8%)
Unemployed	71 (8.4%)	98 (8.8%)
Assumed unemployed (age < 18 or > 65 years)	181 (21.3%)	288 (26.0%)
Missing values	10 (1.2%)	15 (1.4%)
Region ^d^		
Predominantly urban	259 (30.5%)	347 (31.4%)
Intermediate	190 (22.4%)	246 (22.2%)
Predominantly rural	399 (47.0%)	513 (46.3%)
Missing values	0 (0.0%)	1 (0.1%)
Comorbidities ^e^		
0	270 (31.8%)	457 (41.3%)
1	224 (26.4%)	276 (24.9%)
2	121 (14.3%)	145 (13.1%)
3	108 (12.7%)	103 (9.3%)
4	64 (7.5%)	61 (5.5%)
≥5	61 (7.2%)	65 (5.9%)

^a^ Patients are index users of either secukinumab or ustekinumab.

^b^ Income is as per the data from the year before index year of drug use.

^c^ Partner and occupational status are as per the data from the year before index year of drug use; classifications adapted from Statistics Sweden (SCB) https://www.scb.se/en/.

^d^ Region is as per the data from the year before index year of drug use; obtained as per Eurostats Degree of Urbanisation (DEGURBA) classification https://ec.europa.eu/eurostat/web/degree‐of‐urbanisation/background. Predominantly urban category includes Stockholm county, Intermediate category includes Skåne and Västra Götalands counties; all other counties were included in the Predominantly rural category.

^e^ Baseline comorbidities are clinical conditions recorded before index drug use. This includes – (major adverse cardiovascular events (MACE), angina, coronary artery disease (CAD), cancer, immunocompromised status, diabetes, chronic obstructive pulmonary disease (COPD), dyslipidemia, hypertension, HIV, arrhythmias, arthritis, rheumatoid diseases, Crohn's disease, ulcerative colitis, liver diseases, renal diseases, psoriatic arthritis, asthma) before index.

The crude IR per 1000 person‐days for RTI and UTI treated in primary care (antibiotics for RTI and UTI outcome) was 1.10 (95% CI: 0.98‐1.24) among secukinumab users and 0.61 (95% CI: 0.56‐0.66) among ustekinumab users (Table 2); the IRR per 1000 person‐days was 1.80 (95% CI: 1.55‐2.09). For RTI treated in primary care specifically, the IRs per 1000 person‐days were 0.76 and 0.45 for secukinumab and ustekinumab, respectively. For UTI treated in primary care specifically, the IRs per 1000 person‐days were 0.35 and 0.20 for secukinumab and ustekinumab, respectively. The IRRs per 1000 person‐days were 1.68 (95% CI: 1.41‐2.00) and 1.85 (95% CI: 1.44‐2.34) for RTI and UTI treated in primary care. For severe infections comprising of RTI and UTI and for candidiasis diagnosed in specialist care, few events were observed (Table 2).

**TABLE 2:** Number of events and crude incidence rates of RTI, UTI and candidiasis per 1000 person‐days, incidence rate ratios of RTI, UTI and candidiasis per 1000 person‐days and crude and adjusted hazard ratios (HR, Cox proportional hazards model, 95% CI) for secukinumab use and ustekinumab use

Outcome	Secukinumab	Ustekinumab
Antibiotics for RTI and UTI		
Events	270	496
Overall person‐time (1000 person‐days)	244.5	810.6
IR (95% CI)	1.10 (0.98‐1.24)	0.61 (0.56‐0.66)
IRR (95% CI)	1.80 (1.55‐2.09)	Ref
Crude HR	1.32 (1.12‐1.54)	Ref
Adjusted HR	1.22 (1.03‐1.43)	Ref
Antibiotics for RTI		
Events	202	401
Overall person‐time (1000 person‐days)	262.9	879.6
IR (95% CI)	0.76 (0.66‐0.88)	0.45 (0.41‐0.50)
IRR (95% CI)	1.68 (1.41‐2.00)	Ref
Crude HR	1.26 (1.05‐1.51)	Ref
Adjusted HR	1.18 (0.98‐1.42)	Ref
Antibiotics for UTI		
Events	104	214
Overall person‐time (1000 person‐days)	288.9	1100
IR (95% CI)	0.35 (0.29‐0.43)	0.20 (0.17‐0.23)
IRR (95% CI)	1.85 (1.44‐2.34)	Ref
Crude HR	1.27 (0.98‐1.64)	Ref
Adjusted HR	1.13 (0.87‐1.46)	Ref
Severe RTI and UTI		
Events	24	76
Overall person‐time (1000 person‐days)	311.9	1200
IR (95% CI)	0.07 (0.05‐0.11)	0.06 (0.05‐0.07)
IRR (95% CI)	1.21 (0.73‐1.94)	Ref
Crude HR	1.04 (0.63‐1.72)	Ref
Adjusted HR	0.96 (0.57‐1.61)	Ref
Severe RTI		
Events	20	63
Overall person‐time (1000 person‐days)	311.9	1200
IR (95% CI)	0.06 (0.04‐0.09)	0.05 (0.04‐0.06)
IRR (95% CI)	1.22 (0.69‐2.04)	Ref
Crude HR	1.15 (0.66‐2.01)	Ref
Adjusted HR	1.03 (0.58‐1.82)	Ref
Severe UTI		
Events	4	15
Overall person‐time (1000 person‐days)	311.4	1200
IR (95% CI)	0.01 (0.004‐0.03)	0.01 (0.007‐0.02)
IRR (95% CI)	1.02 (0.24‐3.22)	Ref
Crude HR	0.65 (0.20‐2.09)	Ref
Adjusted HR	0.66 (0.19‐2.22)	Ref
Candidiasis		
Events	13	21
Overall person‐time (1000 person‐days)	307.9	1200
IR (95% CI)	0.04 (0.02‐0.07)	0.02 (0.01‐0.03)
IRR (95% CI)	2.41 (1.11‐5.05)	Ref
Crude HR	1.55 (0.74‐3.25)	Ref
Adjusted HR	1.80 (0.84‐3.84)	Ref

*Note:* Crude HR – unadjusted for any variable.

*Note:* Adjusted HR – adjusted for age, sex, education, income, partner/civil status, occupational status, region, (psoriatic arthritis, cancer, immunocompromised status, diabetes, chronic obstructive pulmonary disease (COPD), renal diseases, tobacco use, radiation, bacterial infections, asthma) before index.

We report crude and adjusted analyses using Cox proportional‐hazards models, comparing secukinumab users with ustekinumab users (Table 2). In the adjusted analysis, an increased risk of RTI and UTI treated in primary care (HR: 1.22, 95% CI: 1.03‐1.43) was observed in secukinumab users compared to ustekinumab users. The HRs for RTI and UTI individually were similar, 1.18 (0.98‐1.42) and 1.13 (0.87‐1.46). For severe RTI and UTI diagnosed in specialist care, few events were observed (Table 2). For candidiasis the observed adjusted HR was 1.80 (0.84‐3.84). For the sensitivity analysis restricted to the first 3 years on the market for both drugs, no adjusted HRs reached statistical significance. The antibiotic use adjusted HRs were 0.97 (0.72‐1.30), 0.82 (0.60‐1.12) and 1.48 (0.83‐2.64) for antibiotics for RTI and UTI, antibiotics for RTI only and antibiotics for UTI only, respectively. The adjusted HRs for outcomes based on diagnoses were 2.42 (0.70‐8.40), 2.90 (0.67‐12.53) and 2.12 (0.17‐26.13) for RTI and UTI, RTI only and UTI only, respectively. For candidiasis the observed adjusted HR was 3.91 (0.43‐35.7).

## 4 DISCUSSION

Quantifying safety information by obtaining comparative results from real‐world data is useful to make informed therapeutic decisions for individuals with psoriasis. Our study findings showed secukinumab users with a higher risk of infections treated in primary care compared to ustekinumab users. This is consistent with the results from the 52‐week CLEAR RCT on secukinumab and ustekinumab use, where the safety profiles of both drugs were found to be comparable with a slightly higher proportion of adverse events among the secukinumab group (64.2%) compared to the ustekinumab group (58.3%), with non‐serious infections being the most common adverse events.^41^ Infections in our study were largely driven by the increased risk of RTI treated in primary care, although not statistically significant. Similarly, a previous long‐term pooled safety analysis of secukinumab highlights upper RTI as one of the most common adverse events for secukinumab (3.3%).^22^ As for ustekinumab, a long‐term analysis of ustekinumab demonstrated no elevated risk of infections after 5 years of ustekinumab exposure.^18^ Another large analysis of safety data from clinical trials and post‐marketing surveillance database reports the association of secukinumab with fewer adverse events, with upper RTI being the most frequent.^42^ For severe RTI and UTI observed in our study, there were no significant differences in the risk of infections possibly due to the smaller number of events. Immune dysregulation in psoriasis is known to cause increased production of pro‐inflammatory cytokines. Some of these cytokines (eg, IL‐22, IL‐23, IL‐17) have significant roles in immune defence mechanisms against pathogens such as *Candida albicans*. Therefore, patients treated with IL‐antagonists, are at a higher risk of developing Candida infections.^27^ A study exploring previously conducted clinical trials to measure the risk of candidiasis among patients treated with IL‐targeted therapies, presents 1.7% overall incidence of candidiasis among secukinumab users and 2.3% overall incidence of candidiasis among ustekinumab users.^26^ In our study, for candidiasis treated in hospital care we found a slightly elevated but statistically insignificant risk in secukinumab users as compared to ustekinumab users. The majority of cases were vaginal candidiasis followed by oral candidiasis (data not shown). When restricting the study period to the first 3 years of market availability for both drugs, no differences were found indicating that potential channelling bias had little impact on the main results.

Our study has multiple strengths. Firstly, the study population included all psoriasis patients treated with secukinumab and ustekinumab during the study period in Sweden; minimising the potential for selection bias. Secondly, register‐based data covers people from all regions in Sweden; increasing the generalizability of the findings to Sweden and/or to countries with similar healthcare systems and populations. Thirdly, data on baseline characteristics and clinical comorbidities were collected from linking of multiple registers, to ensure completeness of information and to avoid differential misclassification. In addition to severe RTI and UTI diagnosed from NPR, RTI and UTI treated in primary care were captured using antibiotics use recorded in PDR as proxies. The completeness of pharmacy records is guaranteed as they reflect drug dispensation received by patients. Although prescriptions do not reflect actual medication taking behaviour, they do confirm the occurrence of infections which necessitated antibiotics' prescriptions. Similar to our study, antibiotics' prescriptions have been previously used as proxy measures to capture infections.^43^, ^44^, ^45^ Additionally, to the best of our knowledge, this is the first post‐marketing study comparing the use of secukinumab and ustekinumab and risk of RTI, UTI and candidiasis outside of clinical trials. Also, the use of active comparator design that is, ustekinumab being compared to secukinumab among individuals with the same disease, reduces the potential for confounding by indication.^46^

However, this study has its share of limitations. The ustekinumab group had a longer follow‐up period compared to the secukinumab group. This could potentially introduce bias in the study results providing an overestimation of risk for ustekinumab. A restricted analysis using only data from the first 3 years of market availability was added as a sensitivity analysis. However, it resulted in the loss of all the information from 2012 to 2015 for the ustekinumab group. Individuals in both treatment groups could have received preferential prescriptions of either secukinumab or ustekinumab, based on their relative disease severity. This has potential for channelling bias. Ever‐never exposure definition was used for drug exposure and drug switchers were accounted for only based on their index drug exposure throughout the follow‐up period and not accounted for the switched drug use. This could potentially cause misclassification of exposure. However, the overall proportion of drug switchers during the study period was quite low (~12%, data not shown). Although there were some differences in the baseline comorbidities among the study drug groups, all baseline comorbidities were adjusted for in the final model. Nevertheless, risk for residual confounding and unmeasured confounding (eg, lifestyle factors, body mass index) cannot be ruled out. Also, baseline disease severity of both treatment groups was not accounted for. However, as both groups are treated with biologics, we expect them to have fairly similar severity of disease. Additionally, indications for which the antibiotics were prescribed are unknown. Antibiotic prescription rates can vary between practitioners and across counties in Sweden.^47^, ^48^ Although we have not explored the difference in prescription rates among specific practitioners, we have adjusted for different counties in Sweden. This will capture most of the potential variation. Lastly, our data coverage ended December 2017, since the national Swedish registers are updated annually, for example, NPR is available the earliest in September the next year. Moreover, the process for data application and delivery takes about 1 year. Allowing time for data cleaning, analysis and manuscript writing, we used data available to us early 2019, that is, data availability beyond December 31, 2017 was impossible to obtain.

## 5 CONCLUSION

The study findings provide real‐world evidence on the adverse events – RTI, UTI and candidiasis among secukinumab and ustekinumab users treated for psoriasis and psoriatic arthritis. Specifically, secukinumab users had a slightly increased risk of infections treated in primary care compared to ustekinumab users. Infections are events of clinical significance as they cause varying burden among affected individuals. Continuous, long‐term, comparative safety studies with multiple biologic therapies, among different population groups are needed to validate our study findings and to obtain robust data, which can help develop optimal therapeutic guidelines.

## ETHICS STATEMENT

The study was approved by the Ethical Review Board, Stockholm (reference number 2009/1215‐31/4).

## CONFLICT OF INTEREST

The authors declare no conflicts of interest.

## Supporting information


**Appendix** **S1.** Supporting information.
