# Supplementary material for: Risk of common infections among individuals with psoriasis in Sweden: A nationwide cohort study comparing secukinumab to ustekinumab
Source: Pharmacoepidemiol Drug Saf. 2020 Sep 25;29(12):1562–9. doi: 10.1002/pds.5132 (PMC7756328; doi:10.1002/pds.5132)
Supplement: Supplementary file 1 — Appendix S1. Supporting information. [file PDS-29-1562-s001.docx]

**Appendix**


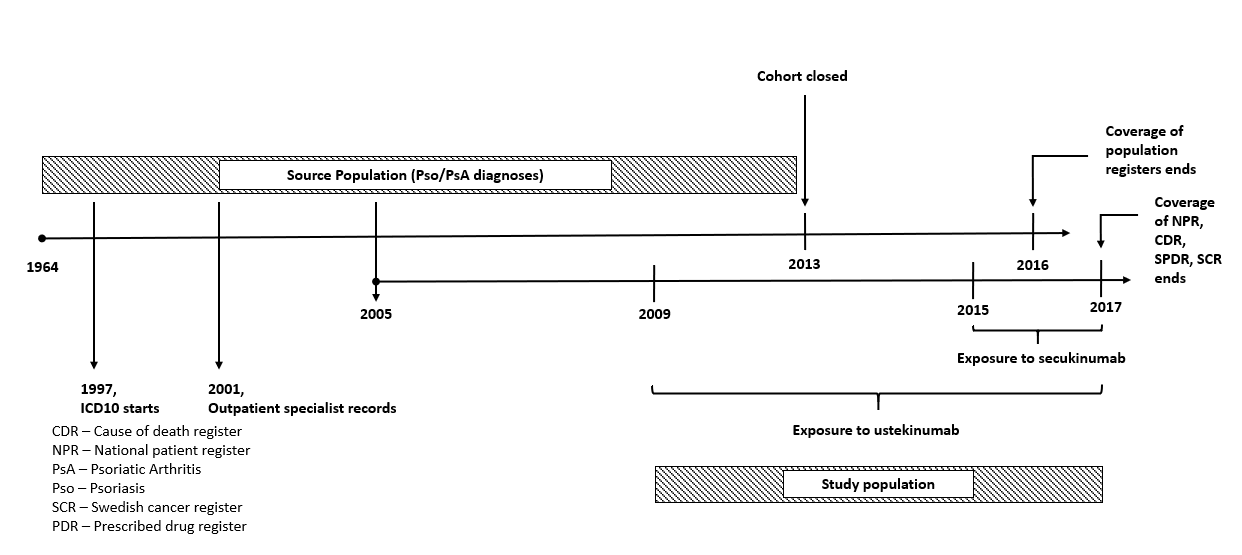


**Figure S1. Cohort chart describing population and follow-up period**

Note. Cohort chart style adapted by - Schneeweiss S, Rassen JA, Brown JS, et al. Graphical Depiction of Longitudinal Study Designs in Health Care Databases. Ann Intern Med. 2019;170(6):398-406. doi:10.7326/M18-3079

The source population includes all individuals registered in the period 1964 – 2013 with psoriasis or psoriasis arthritis.

One individual emigrated before index without a following immigration, i.e. excluded due to uncertain database coverage.

During the study period 2006 - 2017, 1955 individuals fulfilled the criteria of having used the study drugs of interest i.e., either secukinumab or ustekinumab.

Dispensation of ustekinumab or secukinumab during the inclusion period 2009 – 2016.

No dispensation of ustekinumab or secukinumab during the inclusion period 2009 - 2016

**Figure S2. Study flow chart**

| **Table S1. Number of events and crude incidence rates of RTI, UTI and candidiasis per 1000 person-days, incidence rate ratios of RTI, UTI and candidiasis per 1000 person-days and crude and adjusted hazard ratios (HR, Cox proportional hazards model, 95% CI) for secukinumab use and ustekinumab use restricted to the first three years of market availability for secukinumab (2015-2017) and ustekinumab (2009-2011)** | | |
| --- | --- | --- |
| **Outcome** | **Secukinumab** | **Ustekinumab** |
| **Antibiotics for RTI and UTI** | | |
| Events | 270 | 100 |
| Overall person-time (1000 person-days) | 244.5 | 193.7 |
| IR (95% CI) | 1.10 (0.98 – 1.24) | 0.52 (0.42 – 0.63) |
| IRR (95% CI) | 2.14 (1.69 – 2-72) | Ref |
| Crude HR | 1.24 (0.94 – 1.65) | Ref |
| Adjusted HR | 0.97 (0.72 – 1.30) | Ref |
| **Antibiotics for RTI** | | |
| Events | 202 | 90 |
| Overall person-time (1000 person-days) | 262.9 | 208.3 |
| IR (95% CI) | 0.77 (0.67 – 0.88) | 0.43 (0.35 – 0.53) |
| IRR (95% CI) | 1.78 (1.38 - 2.31) | Ref |
| Crude HR | 1.00 (0.74 - 1.35) | Ref |
| Adjusted HR | 0.82 (0.60 - 1.12) | Ref |
| **Antibiotics for UTI** | | |
| Events | 104 | 36 |
| Overall person-time (1000 person-days) | 289.0 | 301.8 |
| IR (95% CI) | 0.36 (0.30 – 0.44) | 0.12 (0.09 – 0.17) |
| IRR (95% CI) | 3.02 (2.05 – 4.54) | Ref |
| Crude HR | 2.10 (1.21 – 3.65) | Ref |
| Adjusted HR | 1.48 (0.83 – 2.64) | Ref |
| **Severe RTI and UTI** | | |
| Events | 24 | 18 |
| Overall person-time (1000 person-days) | 312.0 | 345.1 |
| IR (95% CI) | 0.08 (0.05 – 0.11) | 0.05 (0.03 – 0.08) |
| IRR (95% CI) | 1.48 (0.77 – 2.88) | Ref |
| Crude HR | 3.67 (0.92 – 14.6) | Ref |
| Adjusted HR | 2.42 (0.70 – 8.40) | Ref |
| **Severe RTI** | | |
| Events | 20 | 15 |
| Overall person-time (1000 person-days) | 311.9 | 345.1 |
| IR (95% CI) | 0.06 (0.04 – 0.10) | 0.04 (0.03 – 0.07) |
| IRR (95% CI) | 1.48 (0.72 – 3.10) | Ref |
| Crude HR | 3.67 (0.92 – 14.6) | Ref |
| Adjusted HR | 2.90 (0.67 – 12.53) | Ref |
| **Severe UTI** | | |
| Events | 4 | 3 |
| Overall person-time (1000 person-days) | 311.5 | 344.4 |
| IR (95% CI) | 0.01 (0.00 – 0.03) | 0.01 (0.00 – 0.03) |
| IRR (95% CI) | 1.47 (0.25 – 10.06) | Ref |
| Crude HR | 1.61 (0.15 – 17.61) | Ref |
| Adjusted HR | 2.12 (0.17 – 26.13) | Ref |
| **Candidiasis** | | |
| Events | 13 | 1 |
| Overall person-time (1000 person-days) | 307.9 | 341.8 |
| IR (95% CI) | 0.04 (0.02 – 0.07) | 0.00 (0.00 – 0.02) |
| IRR (95% CI) | 14.4 (2.17 – 613.3) | Ref |
| Crude HR | 3.25 (0.42 – 24,9) | Ref |
| Adjusted HR | 3.91 (0.43 – 35.7) | Ref |
| Crude HR – unadjusted for any variable  Adjusted HR - adjusted for age, sex, education, income, partner/civil status, occupational status, region, (psoriatic arthritis, cancer, immunocompromised status, diabetes, chronic obstructive pulmonary disease (COPD), renal diseases, tobacco use, radiation, bacterial infections, asthma) before index | | |

| **Table S2. ICD-10 codes for respiratory tract infections, urinary tract infections and candidiasis diagnoses and ATC codes for antibiotics used in the treatment of respiratory tract infections and urinary tract infections in primary care** | | |
| --- | --- | --- |
| **Infections** | **ICD-10 Codes** | **Definitions** |
| Respiratory tract infections | J00, J01, J02, J03, J04, J05, J06, J12, J13, J14, J15, J16, J17, J18, J20, J21, J22 | Acute nasopharyngitis, Acute sinusitis, Acute pharyngitis, Acute tonsillitis, Acute laryngitis and tracheitis, Acute obstructive laryngitis and epiglottitis, Acute upper respiratory infections of multiple unspecified sites, Viral pneumonia, Pneumonia due to S. pneumoniae, Pneumonia due to H. influenzae, Bacterial pneumonia, Pneumonia due to other infectious organisms, Pneumonia in diseases classified elsewhere, Pneumonia (organism unspecified), Acute bronchitis, Acute bronchiolitis, Unspecified acute lower respiratory infection |
| Urinary tract infections | N10, N12, N300, N308, N309, N390 | Acute tubulo-interstitial nephritis, Tubulo-interstitial nephritis, Acute cystitis, Other cystitis, Cystitis, Urinary tract infection (site not specified) |
| Candidiasis | B37 | Candidiasis |
| **Antibiotics used for infections treated in primary care** | **ATC Codes** | **Drug names** |
| Respiratory tract infections | J01CE02, J01CA04, J01AA02, J01FA01, J01FA02, J01FA09, J01FA10 | Phenoxymethylpenicillin, amoxicillin, doxycycline, erythromycin, spiramycin, clarithromycin, azithromycin |
| Urinary tract infections | J01CA08, J01EA01, J01EE01, J01MA01, J01MA02, J01XE01 | Pivmecillinam, trimethoprim, sulfamethoxazole and trimethoprim, ofloxacin, ciprofloxacin, nitrofurantoin |

| **Table S3. Frequencies and proportions of comorbidities before index for both drug groups (n,%)** | | |
| --- | --- | --- |
| **Comorbidities** | **Secukinumab** | **Ustekinumab** |
| Major adverse cardiovascular events (MACE)* | 35 (4.1%) | 57 (5.2%) |
| Angina | 32 (3.8%) | 37 (3.3%) |
| Coronary artery disease (CAD) | 30 (3.5%) | 52 (4.7%) |
| Cancer | 26 (3.1%) | 19 (1.7%) |
| Immunocompromised status | 1 (0.1%) | 1 (0.09%) |
| Diabetes | 77 (9.1%) | 117 (10.6%) |
| Chronic obstructive pulmonary disease (COPD) | 18 (2.1%) | 36 (3.3%) |
| Dyslipidaemia | 58 (6.8%) | 88 (7.9%) |
| Hypertension | 169 (19.9%) | 226 (20.4%) |
| HIV | 0 | 0 |
| Arrhythmia | 26 (3.1%) | 25 (2.3%) |
| Arthritis | 209 (24.7%) | 146 (13.2%) |
| Rheumatoid diseases | 128 (15.1%) | 97 (8.8%) |
| Crohn’s disease | 10 (1.2%) | 20 (1.8%) |
| Ulcerative colitis | 15 (1.8%) | 31 (2.8%) |
| Liver diseases | 46 (5.4%) | 71 (6.4%) |
| Renal diseases | 7 (0.8%) | 16 (1.5%) |
| Psoriatic arthritis | 435 (51.3%) | 382 (34.5%) |
| Asthma | 69 (8.1%) | 76 (6.9%) |
| Note: The look-back period for all the comorbidities started from 1 January 1997.  MACE* comprises of acute myocardial infarction, subarachnoid haemorrhage, intracerebral haemorrhage, cerebral infarction and stroke | | |

| **Table S4. Test of proportional-hazards assumption for exposure (secukinumab vs. ustekinumab)** | |
| --- | --- |
| **Outcome** | **p-values (overall, Global test)** |
| Antibiotics for RTI and UTI | 0.56 |
| Antibiotics for RTI | 0.69 |
| Antibiotics for UTI | 0.82 |
| Severe RTI and UTI | 0.67 |
| RTI | 0.94 |
| UTI | 0.46 |
| Candidiasis | 0.22 |
| Interpretation: The p-values were tested with significant level alpha=0.05. Since the p-values for all the study outcomes are greater than the significant level, the proportionality assumption does not fail. | |
